# Serum Epidermal Growth Factor is Low in Schizophrenia and Not Affected by Antipsychotics Alone or Combined With Electroconvulsive Therapy

**DOI:** 10.3389/fpsyt.2020.00104

**Published:** 2020-03-03

**Authors:** Xiaobin Zhang, Wenhuan Xiao, KuanYu Chen, Yaqin Zhao, Fei Ye, Xiaowei Tang, Xiangdong Du

**Affiliations:** ^1^ Institute of Mental Health, Suzhou Psychiatric Hospital, The Affiliated Guangji Hospital of Soochow University, Suzhou, China; ^2^ School of Mental Health, Jining Medical University, Jining, China; ^3^ Department of Psychiatry, Affiliated WuTaiShan Hospital of Medical College of Yangzhou University, Yangzhou, China; ^4^ Department of Nursing, Huzhou University, Huzhou, China; ^5^ Nanjing Brain Hospital, Nanjing Medical University, Nanjing, China

**Keywords:** schizophrenia, epidermal growth factor, electroconvulsive therapy, antipsychotics, clinical response

## Abstract

**Background:**

Epidermal growth factor (EGF) is implicated in the pathogenesis of schizophrenia, suggesting possible value as a biomarker for disease severity or treatment response. However, basal EGF levels and changes during treatment are inconsistent across studies. The goal of this study is to compare serum EGF in schizophrenia patients before and after treatment with antipsychotics alone or combined with electroconvulsive therapy (ECT).

**Method:**

Patients meeting DSM-IV diagnostic criteria for schizophrenia were recruited from June 2013 to December 2015 (n = 186) and followed up after 8 weeks of treatment with antipsychotics alone (n = 119, drug group) or combined with ECT (n = 67, ECT group). Serum EGF levels were measured by ELISA and compared among patients and 74 healthy control subjects. Psychopathology and clinical effects were assessed using the Positive and Negative Syndrome Scale (PANSS).

**Results:**

Basal serum EGF was significantly lower in the entire patient cohort compared to healthy controls (*P* < 0.05). Repeated-measures ANOVA showed no main effect of time (*F* = 1.273; *P* = 0.261), time × group interaction (*F* = 1.228; *P* = 0.270), main effect of clinical response (*F* = 0.191; *P* = 0.663), or group × clinical interaction (*F* = 1.765; *P* = 0.186) on serum EGF. Serum EGF levels did not change significantly following antipsychotic drug or combined therapy (*P* > 0.05). Additionally, neither basal EGF nor EGF change was associated with the clinical response to drug or combined treatment (*P* > 0.05). However, baseline serum EGF was weakly associated with PANSS positive score (pretreatment: *r* = 0.206, posttreatment: *r* = 0.201) and general symptom score (pretreatment: *r* = −0.244). Serum EGF was also associated with duration of illness (pretreatment: *r* = 0.285, posttreatment: *r* = −0.231).

**Conclusions:**

Serum EGF concentration is low in schizophrenia but is unchanged following treatment with antipsychotics alone or combined with ECT, regardless of clinical response. Thus, serum EGF is not a surrogate biomarker for treatment response and is unlikely to be involved in the therapeutic mechanisms of antipsychotics or ECT.

## Introduction

The cytokine epidermal growth factor (EGF) has been proposed as an important effector molecule in schizophrenia (1–3). Dysregulation of dopaminergic signaling is implicated in the expression of schizophrenic symptoms, and it is well documented that EGF signaling regulates dopaminergic neuron activity and monoamine metabolism in the brain (2, 4–9). EGF exerts neuroprotective effects and influences synaptic transmission by binding to the high-affinity EGF receptor (EGFR, also known as ErbB-1 or HER1) (10). In addition, related ErbB receptors may enhance the antidopaminergic actions implicated in the therapeutic mechanism of antipsychotic drugs. Recent studies have reported the abnormal expression of EGF in Parkinson's disease (11), autism (12, 13), brain injury and limb fracture (14), gastric cancer (15), hepatocellular carcinoma (16, 17), psoriasis (18), endometrial carcinoma (19), and primary ovarian cancer (20). Thus, we speculated that abnormal expression of EGF may contribute to schizophrenia symptoms and (or) treatment efficiency.

A *postmortem* study of 32 individuals with schizophrenia found decreased EGF concentrations in the prefrontal cortex and striatum (21) and elevated concentrations in prefrontal cortex compared to matched controls. Several clinical studies have also examined alterations of serum EGF in schizophrenia patients (21–23) but have yielded discrepant results. Two studies reported that peripheral EGF levels were substantially higher in patients with schizophrenia compared to healthy controls (22, 23), while others reported below-normal serum levels in patients (21, 24), and still others found no difference between patients and controls (25, 26). Haring and colleagues (23) explored the impact of antipsychotic drug treatment on serum EGF in 38 schizophrenia patients before and after 7 months of medical intervention and found significantly elevated serum EGF in first-episode schizophrenia patients compared to controls and below normal levels following treatment. Moreover, the magnitude of the decrease correlated with the suppression of positive symptoms, suggesting that serum EGF reduction contributes to the clinical response. Conversely, Futamura (21) found that 6 months of antipsychotic treatment (haloperidol) had no effect on EGF protein concentrations in male Wistar rats. Therefore, the contributions of central and peripheral EGF to disease pathogenesis and treatment response remain obscure.

In a previous study, we measured serum brain derived neurotrophic factor (BDNF) in 160 schizophrenia patients between the ages of 16 and 65 before and after treatment with antipsychotic agents and ECT, and we found that both treatments increased serum BDNF (27). Many studies have examined BDNF levels in schizophrenia and revealed that BDNF influences the therapeutic mechanisms of ECT (28–30). However, no studies to date have determined whether EGF also contributes to therapeutic effect of ECT in schizophrenia.

Therefore, this study was designed to test whether serum EGF levels are associated with symptom severity at baseline or with treatment response to antipsychotics administered alone or combined with ECT. We hypothesized that basal EGF levels are significantly lower in patients compared to healthy controls and increase during treatment concomitant with suppression of symptoms.

## Method

### Subjects

224 patients with schizophrenia were enrolled between June 2013 and December 2015 from an inpatient clinic at WuTaiShan Hospital, Yangzhou, China. The diagnosis of schizophrenia was confirmed by experienced psychiatrists according to the DSM-IV criteria. Patients were recruited according to the following inclusion criteria: (1) age 18 to 60 years and (2) no prior treatment with antipsychotic medications or free of oral antipsychotics for at least 2 weeks before the study began. 74 healthy volunteers were recruited through local advertisements. Control subjects were screened to rule out psychiatric disorders using the General Health Questionnaire. Patients and controls with intellectual disability, organic brain disorders, physical diseases or recent history of alcohol and/or substance abuse/dependence (within past 5 years) were excluded. This study was approved by the Ethics Committee of Yangzhou WuTaiShan Hospital. Written informed consent was obtained from all subjects after a complete description of the study methods and goals.

### Procedure

**Figure 1:**
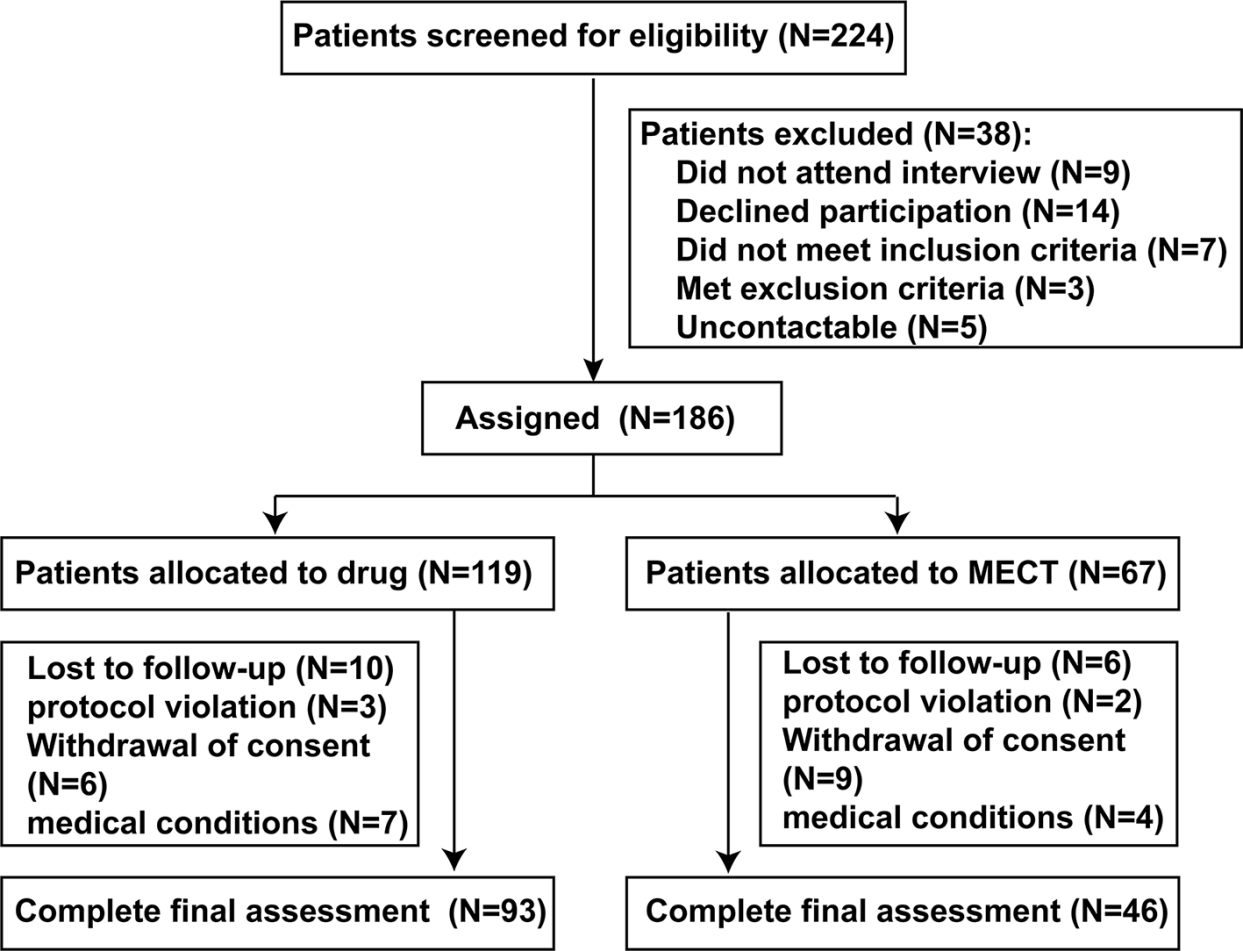
Diagram for patients with schizophrenia.

Of the 224 participants initially screened, 186 (89 drug-naive and 97 drug-free for at least two weeks) elected to take part in the trial. As this is a naturalistic longitudinal study, the research team did not interfere on the patients' care and treatment. The method of treatment was determined by the judgment of the chief psychiatrist based on clinical characteristics of the patient with schizophrenia. Patients were divided into two groups: the ECT + antipsychotics group (‘ECT group’, n = 67) and the antipsychotic agents only group (‘drug group’, n = 119). Although electroconvulsive therapy (ECT) has generally been reserved for patients' refractory to other forms of treatment, it is used as a first-line therapy for schizophrenia under certain conditions: (a) excitement and (or) uncooperativeness, (b) psychotic features, (c) hostility and poor impulse control, (d) refusal to eat and drink, or (e) self-blame, self-injury, or suicidal behavior. After 8 weeks of follow-up, 47 patients failed to complete follow-up assessment, so the data from 139 patients (74.7%) were included in the final analysis. There was no significant difference in the rate of study completion between the drug and ECT groups (*P* > 0.05) (**Figure 1**).

We also investigated the administration of psychotropic medications to these patients. One hundred and eight individuals (58.1%) of the schizophrenic patients were on treatment with a single antipsychotic agent, with 94 subjects receiving atypical antipsychotics and 14 receiving typical antipsychotic treatment. In detail, 21 patients received risperidone, 16 received clozapine and 15 received olanzapine. 13 received quetiapine, 12 received aripiprazole, 10 received amisulpride, 7 received ziprasidone, 7 received chlorpromazine, five received sulpiride, and the remaining 2 received perphenazine. The doses of antipsychotics were adjusted based on psychiatric symptoms. Average daily doses were expressed in chlorpromazine equivalents using defined daily dosages. Furthermore, seventy-eight patients (41.9%) were prescribed more than one antipsychotic drug concurrently. Moreover, Patients were not treated with depot antipsychotics.

A blinded clinical evaluation team completed Positive and Negative Syndrome Scale (PANSS) ratings before and after treatment. Inter-rater reliability coefficients for PANSS scores exceeded 0.90. Treatment-resistant schizophrenia (TRS) was defined by at least two failed adequate trials with different antipsychotics (at chlorpromazine-equivalent doses of ≥600 mg/day for ≥6 consecutive weeks) (31). Patients were divided into response and nonresponse subgroups. Definition of treatment response based on a 50% reduction of PANSS total scores for acutely ill, 25% for refractory patients (32). In total, 27 patients (19.9%) met the TRS criteria: 8 patients in drug group and 19 in the ECT group.

### ECT Procedure

Participants signed separate informed consent for ECT. All subjects' medical histories and current physical, neurological, and laboratory examination results were evaluated to determine the general medical condition prior to undergoing ECT. In all cases, ECT was performed with bilateral placement using the Thymatron-DGx device (Somatics, Lake Bluff, Ill.) under anesthesia with thiopental or S-ketamine and succinylcholine administration for muscle relaxation. The ECT procedure has been described in detail elsewhere (27). ECT was administered three times per week (mean number: 6.79 ± 1.69, range: 6–10). The total number of ECT sessions for each patient was determined by the authors' consensus opinion based on clinical improvement and adverse side effects.

### Serum Epidermal Growth Factor Determination

Blood was collected from the subjects at 7:00 am before breakfast into anticoagulant-free tubes. Serum was isolated by centrifugation at 3,000 g for 10 min and then stored at −80°C until analysis. Serum EGF levels were measured by a sandwich ELISA method using the EGF Emax ImmunoAssay System (R&D Systems, Minneapolis, MN, USA) after appropriate dilution with the Block and Sample solutions. All assays were performed according to the manufacturer's instructions. The intra- and interassay precisions were within 5–10%.

### Statistical Analyses

All analyses were performed by two researchers blinded to the sample collection and treatment status. Data was analyzed using IBM SPSS statistics 23.0 software (IBM Corporation, Armonk, NY, USA). All group data are expressed as mean ± standard deviation or frequency. Data sets that were not normally distributed were log-transformed. Group differences in demographic parameters, clinical data, and serum EGF were compared by T-test, analysis of variance (ANOVA), or Chi-square test as indicated. For statistical comparison of EGF levels and psychotic symptoms between drug and ECT groups, we tested the main effect of group (drug *vs.* ECT), main effect of time pretreatment and posttreatment, and group × time interaction effect on EGF levels using repeated measures ANOVA. Thereafter, the changes in serum EGF levels from baseline to the endpoint for responders *vs.* nonresponders (base on criteria defined above) were assessed.

Pearson correlation coefficients were calculated to test the relationships between demographic variables, PANSS, and serum EGF. Last, as a follow-up to these results, linear regression analysis was used to examine the relationship between serum EGF concentration and the main effect among schizophrenia patients, with age, BMI, and sex as covariates. We applied Bonferroni correction for multiple comparisons. Statistical significance was set at a *P* value of <0.0167 (Bonferroni correction *P* = 0.05/3).

## Results

### Lower Baseline and Posttreatment Serum Epidermal Growth Factor in Schizophrenia Patients Versus Healthy Controls

Sociodemographic and clinical data for each group are summarized in **Table 1**. There were no significant differences in mean age, sex ratio, years of education, or smoking status among the drug, ECT, and control groups (*P* > 0.05). However, ANOVA revealed a significantly higher BMI in the patient groups relative to the control group at baseline (*F* = 3.230; *P* = 0.042). Among those who completed the study were 66 first-episode schizophrenia patients, 59 with multiple episode psychosis with acute exacerbation, and 14 with chronic stable schizophrenia. There was a significant difference in clinical state between drug group and ECT group (*x* ^2^ = 13.465; *P* = 0.001). In addition, the drug group had fewer refractory patients than the ECT group (*x* ^2^ = 21.029; *P* = 0.001).

**Table 1:** Demographics of participants.

Baseline characteristics	drug group (n = 93)	ECT group (n = 46)	Control group (n = 74)	*P*-value
Age (years)	33.29 ± 8.43	31.98 ± 7.04	34.85 ± 11.27	0.242
Sex (n, %)	49 (52.7%)	25 (54.3%)	41/33	0.939
Education (years)	10.57 ± 3.58	11.48 ± 3.47	11.22 ± 3.09	0.260
Smokers(n, %)	38 (40.9%)	28 (60.9%)	31/43	0.062
BMI (kg/m^2^)	24.56 ± 3.99	23.98 ± 3.98	22.99 ± 3.97	0.042
Duration of illness (months)	13.01 ± 9.47	14.41 ± 8.76	–	0.401
Family history of mental disorders (n, %)	21 (22.6%)	12 (26.1%)	–	0.648
Clinical state	–	–	–	0.001
First-episode (n, %)	50(53.8%)	16(34.8%)	–	–
Multiple episode psychosis with acute exacerbation (n, %)	30(32.2%)	29(63.0%)	–	–
Chronic stable schizophrenia (n, %)	13(14.0%)	1(2.2%)	–	–
Refractory patient(n, %)	8(8.6%)	19(41.3%)	–	0.001

Levels of serum EGF (log-transformed) are displayed in **Table 2**. Baseline serum EGF was significantly lower in patients than in controls (1.87 ± 0.05 *vs.* 1.98 ± 0.04) but did not differ between the drug group (1.88 ± 0.07) and ECT group (1.86 ± 0.07) (*P* > 0.05). Moreover, the serum EGT of the patients after treatment was still lower than that of the control group. However, there was no difference between the treatment groups (drug group: 1.88 ± 0.07; ECT group: 1.87 ± 0.04). Additionally, serum EGF did not differ significantly between male and female patients either before or after treatment (before: *t* = 0.284; *P* = 0.777; after: *t* = 0.926; *P* = 0.355).

**Table 2:** Reports the descriptive and inferential statistics, of PANSS and EGF level, separated by Time (pre- *vs.* postassessment) and Group (drug- *vs.* ECT group).

Dimensions	Assessment				Statistical analyses		
	Preassessment		Postassessment		Factor time	Factor group	Time by group interaction
	Drug group	ECT group	Drug group	ECT group	*F (P)*	*F (P)*	*F (P)*
PANSS-*P*	23.69 ± 5.98	24.07 ± 5.74	15.82 ± 5.27	16.46 ± 5.44	*F* = 209.641 P = 0.000	*F* = 0.349 P = 0.556	*F* = 0.060 P = 0.807
PANSS-N	22.59 ± 5.43	29.07 ± 5.49	13.55 ± 3.97	13.46 ± 3.38	*F* = 612.597 P = 0.000	*F* = 21.781 P = 0.000	*F* = 43.455 P = 0.000
PANSS-G	30.43 ± 5.61	29.52 ± 5.53	19.91 ± 6.37	18.98 ± 6.37	*F* = 285.908 P = 0.000	*F* = 1.093 P = 0.298	*F* = 0.001 P = 0.983
PANSS-TOT	76.71 ± 7.66	82.65 ± 7.54	49.28 ± 11.07	48.89 ± 10.98	*F* = 915.172 P = 0.000	*F* = 4.059 P = 0.046	*F* = 9.796 P = 0.002
Log EGF	1.88 ± 0.07	1.86 ± 0.07	1.88 ± 0.07	1.87 ± 0.04	*F* = 1.273 P = 0.261	*F* = 4.609 P = 0.054	*F* = 1.228 P = 0.270

PANSS, positive and negative syndrome scale; EGF, epidermal growth factor.

### Serum Epidermal Growth Factor was not Altered by Treatment

Patients, in particular ECT groups, had more severe negative symptoms and higher mean baseline PANSS total scores. Furthermore, patients were divided into two groups based on the reduction in PANSS score following treatment. Seventy-one patients (76.3%) were classified as responders in the drug group, and 38 (82.6%) in the ECT group, and the therapeutic response rate did not differ between the drug and ECT group (*x* ^2^ = 0.714; *P* = 0.398).

Repeated-measures ANOVA showed no main effect of time (*F* = 1.273; *P* = 0.261), time × group interaction (*F* =1.228; *P* =0.270), main effect of clinical response (*F* = 0.191; *P* = 0.663), or group × clinical interaction (*F* = 1.765; *P* = 0.186) on serum EGF. Serum EGF values were similar regardless of clinical response and (or) group allocation (**Table 2**). Furthermore, repeated-measures-ANOVA revealed no influence of any sociodemographic and clinical factor, such as age, sex, BMI, and smoking status on serum EGF change following treatment (*P* > 0.05).

The effect of antipsychotics on EGF levels was also examined, and no significant differences were observed in the concentrations of serum EGF between the patients taking typical and atypical antipsychotic drugs or single-agent and combination therapy with antipsychotics (*P* > 0.05). In addition, there was no correlation between serum EGF levels and dose of antipsychotic drugs prescribed for in-patients (*P* > 0.05).

### Serum Epidermal Growth Factor Levels Were Weakly Associated With Clinical Symptoms

**Table 3** presents the correlations between age, duration of illness, PANSS scores pretreatment and posttreatment, and log serum EGF values (both pretreatment and posttreatment). Baseline serum EGF was weakly associated with PANSS positive score (pretreatment: *r* = 0.206, posttreatment: *r* = 0.201) and general symptom score (pretreatment: *r* = −0.244). Serum EGF was also associated with duration of illness (pretreatment: *r* = 0.285, posttreatment: *r* = −0.231). However, alterations in PANSS scores were not associated with alterations in serum EGF levels for the entire patient cohort (*P* > 0.05). Stepwise multiple regression analysis showed that baseline serum EGF did not predict the improvement of psychiatric symptoms after ECT or antipsychotics (*P* > 0.05).

**Table 3:** Correlation EGF levels and demographic variables and clinical symptoms.

		1	2	3	4	5	6	7	8	9	10
1	Age (years)	–	.184*	.008	.090	−.058	.058	.216*	.115	−.106	.012
2	Duration of illness		–	.043	.186*	.164	.089	−.111	.061	.285**	−.231**
3	PANSS-*P* (pre)			–	.429**	−.151	−.162	−.183*	−.096	.206*	−.013
4	PANSS-*P* (post)				–	−.067	.158	.058	.171*	.201*	−.023
5	PANSS-N (pre)					–	.278**	−.191*	.075	−.057	−.140
6	PANSS-N (post)						–	−.048	.258**	−.106	-.036
7	PANSS-G (pre)							–	.294**	−.244**	.097
8	PANSS-G (post)								–	−.152	−.072
9	Log EGF (pre)									–	−.013
10	Log EGF (post)										–

PANSS, positive and negative syndrome scale; EGF, epidermal growth factor; pre, preassessment; post, postassessment; *P < .05; **P < .01.

## Discussion

In this short-term preliminary clinical study, we found lower serum EGF in a cohort of schizophrenia patients compared to a healthy control group. However, serum EGF remained lower after treatment with antipsychotics (alone or with ECT). Moreover, neither baseline serum EGF nor posttreatment serum EGF differed between treatment responders and nonresponders. Thus, serum EGF is not a biomarker for treatment response.

Compared to the healthy control group, serum EGF was significantly lower in patients prior to drug or combined drug + ECT treatment, in agreement with several previous reports of lower serum EGF in both acute and chronic schizophrenia patients (21, 24). EGF regulates cell growth, proliferation, and differentiation through binding to high-affinity EGFRs (1, 4, 9). Accumulating evidence suggests that neurotrophic factors and cytokines mutually interact to influence neurogenesis and transmitter release, both of which are disrupted in schizophrenia. Low basal serum EGF suggests dysregulation of neuroprotective and inflammatory responses in schizophrenia.

In addition, serum EGF did not change significantly after drug or combined treatment. Similarly, Futamura (21) found that chronic treatment with haloperidol, a typical antipsychotic drug, had no effect on brain or serum EGF levels in a rat model of schizophrenia (21). However, our findings are at odds with the results by Haring et al. (23), which suggests that EGF protein levels were elevated in first-episode psychosis patients compared to controls and appears to be reduced by antipsychotic drug treatment (23). We speculate these differences may be associated with variations in antipsychotic dosages, drug species, total treatment duration, detection methods, or the symptom profile of the enrolled patients among studies.

We also assessed the therapeutic effect of antipsychotic monotherapy and combined antipsychotics plus ECT in schizophrenia patients. Both interventions improved psychiatric symptoms without significant difference as assessed by the PANSS. In addition, we found no relationship between baseline EGF or treatment-dependent change and clinical response. However, the serum levels of EGF at baseline were correlated with clinical parameters such as duration of illness and PANSS scale score, consistent with previous studies (25) reporting associations between EGF and the Brief Psychiatric Rating Scale (BPRS) scores. Despite lack of effect of treatment or of improvement in responders on EGF levels, there was a correlation between PANSS scores and serum EGF levels, indicating that EGF may serve as an indicator of severity of disease instead of therapeutic mechanisms of antipsychotics and ECT in patients with schizophrenia. However, the cause–effect relationship between psychiatric symptoms and EGF levels requires further investigation; one possibility is that deficient neurogenesis and chronic inflammatory responses due to EGF deficiency contribute to some symptoms.

On the basis of a previous work (2, 9, 13, 26), we expected that serum levels of EGF protein concentrations are abnormally low in patients with schizophrenia patients before treatment compared with control subjects, and antipsychotics or ECT brings about their beneficial effects by altering levels of EGF. Results of this study provide evidence that abnormal levels of EGF may be implicated in the etiology of schizophrenia, which partly supported our hypothesis. However, we could not find any significant changes in serum levels of EGF in patients with schizophrenia after antipsychotics alone or combined with ECT. It is possible that blood EGF measurement will not represent a surrogate biomarker for treatment response as no statistically significant differences were present between the response and no response. Alternatively, it is unlikely that EGF was involved in the therapeutic mechanisms of antipsychotics or ECT, of which the pathogenesis is still unclear. Animal research has showed unchanged EGF levels in the brain or serum following long-term administration of typical antipsychotic agents haloperidol, which adds to the evidence that antipsychotics and ECT would not improve psychiatric symptoms *via* increasing EGF levels in adults with schizophrenia.

Several limitations of the present research should be noted. First, the small number of enrolled patients limited statistical strength and precluded analysis of possible differences between subgroups defined by various clinical and demographic characteristics (aside from sex). Large-scale studies are still warranted to assess such potential differences. Moreover, participant discontinuation may have introduced selection bias. Third, it is unclear whether and how peripheral EGF can pass through the blood–brain barrier. Thus, the relationship between peripheral and central EGF concentration is uncertain. Further, the precise relationships among peripheral EGF, central EGF in relevant brain regions, and specific symptoms have not been established. Nonetheless, the current findings suggest that peripheral EGF is of little value for predicting or gauging therapeutic response.

In conclusion, schizophrenia patients have significantly lower baseline serum EGF levels than healthy controls even after obvious symptomatic improvement using antipsychotic drugs alone or drugs plus ECT. Larger-scale studies are required for confirmation and to assess potential differences in baseline EGF or changes during treatment among clinical subgroups.

## Data Availability Statement

The datasets generated for this study will not be made publicly available due to ethical restrictions. The datasets can be requested from the corresponding authors upon reasonable requests.

## Ethics Statement

The studies involving human participants were reviewed and approved by the Ethics Committee of Yangzhou WuTaiShan Hospital. The patients/participants provided their written informed consent to participate in this study.

## Author Contributions

WX, XZ, and XD were responsible for the study design, statistical analysis, and manuscript preparation. WX, KC, YZ, FY, and XT were responsible for recruiting the subjects and collecting clinical data and performing the clinical rating. XZ, XD, and XT were involved in writing the protocol, providing the funding for the study. All authors contributed to and have approved the final manuscript.

## Funding

Suzhou Key Medical Center for Psychiatric Diseases (Szzx201509). The Scientific and Technological Program of Suzhou (SS201752, SS201706). Introduction Project of Suzhou Clinical Expert Team (SZYJTD201715). Medical scientific research project of Jiangsu Provincial Commission of Health and Family Planning (No. H2018041). Medical youth talent projects in Jiangsu Province (No. QNRC2016314). Key Diagnosis and treatment Program of Suzhou (LCZX201919). Postgraduate Research & Practice Innovation Program of Jiangsu Province (No. KYCX18_1497). The finding sources of this study had no role in study design, data collection and analysis, decision to publish, or preparation of the article.

## Conflict of Interest

The authors declare that the research was conducted in the absence of any commercial or financial relationships that could be construed as a potential conflict of interest.
